# Structural Insight into the Resistance of the SARS-CoV-2 Omicron BA.4 and BA.5 Variants to Cilgavimab

**DOI:** 10.3390/v14122677

**Published:** 2022-11-29

**Authors:** Shigeru Fujita, Yusuke Kosugi, Izumi Kimura, Daichi Yamasoba, The Genotype to Phenotype Japan (G2P-Japan) Consortium, Kei Sato

**Affiliations:** 1 Division of Systems Virology, Department of Microbiology and Immunology, The Institute of Medical Science, The University of Tokyo, Tokyo 1088639, Japan; 2 Graduate School of Medicine, The University of Tokyo, Tokyo 1130033, Japan; 3 Faculty of Medicine, Kobe University, Kobe 6500017, Japan; 4 International Research Center for Infectious Diseases, The Institute of Medical Science, The University of Tokyo, Tokyo 1088639, Japan; 5 International Vaccine Design Center, The Institute of Medical Science, The University of Tokyo, Tokyo 1088639, Japan; 6 Graduate School of Frontier Sciences, The University of Tokyo, Kashiwa 2778561, Japan; 7 Collaboration Unit for Infection, Joint Research Center for Human Retrovirus Infection, Kumamoto University, Kumamoto 8600811, Japan; 8 CREST, Japan Science and Technology Agency, Kawaguchi 3320012, Japan

**Keywords:** SARS-CoV-2, COVID-19, Omicron, BA.4, BA.5, cilgavimab, L452R, immune resistance, therapeutic monoclonal antibody

## Abstract

We have recently revealed that the new SARS-CoV-2 Omicron sublineages BA.4 and BA.5 exhibit increased resistance to cilgavimab, a therapeutic monoclonal antibody, and the resistance to cilgavimab is attributed to the spike L452R substitution. However, it remains unclear how the spike L452R substitution renders resistance to cilgavimab. Here, we demonstrated that the increased resistance to cilgavimab of the spike L452R is possibly caused by the steric hindrance between cilgavimab and its binding interface on the spike. Our results suggest the importance of developing therapeutic antibodies that target SARS-CoV-2 variants harboring the spike L452R substitution.

## 1. Introduction

For the prevention of SARS-CoV-2 infection and COVID-19 treatment, a variety of therapeutic monoclonal antibodies (mAbs) have been developed [1]. These therapeutic mAbs bind to the spike protein of SARS-CoV-2 and impair viral entry into the cells [1]. However, substitutions in the viral spike protein potentially affect the efficacy of therapeutic mAbs [1,2,3]. SARS-CoV-2 has considerably diversified during the pandemic, and as of July 2022, the Omicron BA.2 variant is the most dominant variant worldwide [4]. Compared with an ancestral Wuhan/Hu-1/2019 reference strain [5], the Omicron BA.2 variant possesses 29 substitutions in the spike protein [4] and exhibits robust resistance to multiple therapeutic mAbs [6].

Recently, new Omicron sublineages, such as BA.4, and BA.5, emerged in multiple countries and have begun to outcompete BA.2 [7]. The spike proteins of BA.4 and BA.5 are identical, and the BA.4/5 spike bears four substitutions compared with the BA.2 spike. Importantly, we have recently demonstrated that BA.4/5 is resistant to cilgavimab, a therapeutic mAb, and the resistance to cilgavimab of the BA.4/5 spike is attributed to the L452R substitution [8]. Cilgavimab (AZD1061) is used with Tixagevimab (AZD8895) as the antibody cocktail Evusheld (AZD7442) for COVID-19 treatment [9]. Previous reports show that the epitopes of cilgavimab are the residues 440-452 and 490-500 of SARS-CoV-2 spike protein, and these two regions are included in the receptor-binding domain (RBD) [9,10]. Interestingly, although cilgavimab neutralizes a variety of variants of concern and variants of interest, the Epsilon variant (B.1.429) is relatively resistant to cilgavimab [9,11]. Since the common substitution in the Epsilon and BA.4/5 spikes is L452R, it is suggested that the L452R is crucial for the resistance to cilgavimab. However, how the spike L452R substitution renders resistance to cilgavimab remains unclear.

## 2. Materials and Methods

### 2.1. Cell Culture

HEK293T cells (a human embryonic kidney cell line; ATCC CRL-3216) and HOS-ACE2/TMPRSS2 cells (kindly provided by Dr. Kenzo Tokunaga) [12], a derivative of HOS cells (a human osteosarcoma cell line; ATCC CRL-1543) stably expressing human ACE2 and TMPRSS2, were maintained in Dulbecco’s modified Eagle’s medium (high glucose) (Wako, Cat# 044-29765) containing 10% fetal bovine serum (Sigma-Aldrich Cat# 172012-500ML), 100 units penicillin, and 100 μg/mL streptomycin (Sigma-Aldrich, Cat# P4333-100ML).

### 2.2. Plasmid Construction

A plasmid expressing cilgavimab was prepared in our previous study [8]. Plasmids expressing the SARS-CoV-2 spike proteins of Omicron BA.2 and Omicron BA.2 L452R were prepared in our previous studies [4,8]. Plasmids expressing the spike proteins of Omicron BA.2 L452A and BA.2 L452K were generated by site-directed overlap extension PCR using pC-SARS2-S BA.2 [4] as the template and the following primers: pC-S_L452A-F, CAA CTA CAA CTA CGC CTA CAG ACT GTT CA, pC-S_L452A-R, TGA ACA GTC TGT AGG CGT AGT TGT AGT TG, pC-S_L452K-F, GGA GGC AAC TAC AAC TAC AAG TAC AGA CTG TTC AGG AAG AG and pC-S_L452K-R, CTC TTC CTG AAC AGT CTG TAC TTG TAG TTG TAG TTG CCT CC. The resulting PCR fragment was subcloned into the KpnI-NotI site of the pCAGGS vector using an In-Fusion^®^ HD Cloning Kit (Takara, Cat# Z9650N). Nucleotide sequences were determined by DNA sequencing services (Eurofins), and the sequence data were analyzed by Sequencher v5.1 software (Gene Codes Corporation).

### 2.3. Preparation of Monoclonal Antibodies

Cilgavimab was prepared as previously described [4,8]. Briefly, the pCAGGS vectors containing the sequences encoding the immunoglobulin heavy and light chains were cotransfected into HEK293T cells at a 1:1 ratio using PEI Max (Polysciences, Cat# 24765-1). The culture medium was refreshed with Dulbecco’s modified Eagle’s medium (low glucose) (Wako, Cat# 041-29775) containing 10% fetal bovine serum without antibiotics. At 96 h posttransfection, the culture medium was harvested, and the antibody was purified using a NAb protein A plus spin kit (Thermo Fisher Scientific, Cat# 89948) according to the manufacturer’s protocol.

### 2.4. Neutralization Assay

Pseudoviruses were prepared as previously described [4,12]. Briefly, lentivirus (HIV-1)-based, luciferase-expressing reporter viruses were pseudotyped with the SARS-CoV-2 spikes. HEK293T cells (1 × 10^6^ cells) were cotransfected with 1 μg psPAX2-IN/HiBiT [13] 1 μg pWPI-Luc2 [13] and 500 ng plasmids expressing parental S or its derivatives using PEI Max (Polysciences, Cat# 24765-1) according to the manufacturer’s protocol. Two days post transfection, the culture supernatants were harvested and centrifuged. The pseudoviruses were stored at –80 °C until use.

Neutralization assays were performed as previously described [4]. Briefly, the SARS-CoV-2 spike pseudoviruses (counting ~20,000 relative light units) were incubated with serially diluted monoclonal antibodies at 37 °C for 1 h. Pseudoviruses without a monoclonal antibody were included as controls. Then, a 40 μL mixture of pseudovirus and serum was added to HOS-ACE2/TMPRSS2 cells (10,000 cells/50 μL) in a 96-well white plate. Two days post infection, the infected cells were lysed with a Bright-Glo luciferase assay system (Promega, Cat# E2620), and the luminescent signal was measured using a GloMax explorer multimode microplate reader 3500 (Promega). The assay was performed in triplicate, and the 50% inhibitory concentration (IC50) was calculated using Prism 9 (GraphPad Software v9.4.1).

### 2.5. Protein Structure

**Figure 1:**
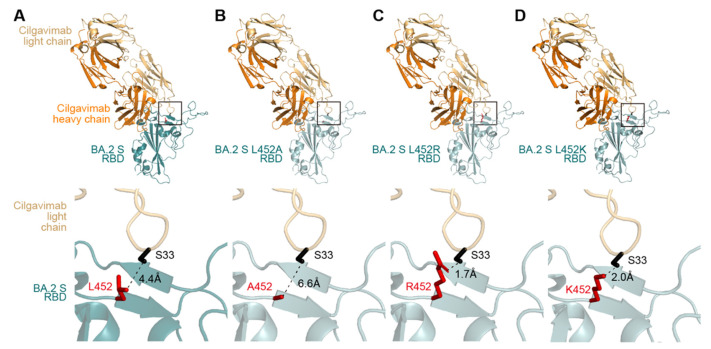
Structural insights of the complex of cilgavimab and the SARS-CoV-2 spike RBD of BA.2 (**A**), BA.2 L452A (**B**), BA.2 L452R (**C**), and BA.2 L452K (**D**). The squared regions are enlarged in the bottom panels. The residue at position 452 of each BA.2 spike RBD mutant is shown in red, while the serine residue at position 33 (S33) of the cilgavimab light chain is shown in black. The distance between these two residues is shown in the panels.

Homology modeling was performed using Discovery Studio 2021 (Dassault Systèmes BIOVIA). In Figure 1, the crystal structure of the SARS-CoV-2 spike receptor binding domain (RBD) (PDB: 7L7E) [9] was used as the template, and 40 homology models of the SARS-CoV-2 spike RBD of the BA.2 mutants were generated using Build Homology Model protocol MODELLER v9.24 [14]. Evaluation of the homology models was performed using PDF total scores and DOPE scores, and the best model for the spike RBD of BA.2 mutants was selected. Other protein structural analyses were performed using the PyMOL Molecular Graphics System, Version 2.5.0 Schrödinger, LLC. In Figure 1, the crystal structure of the complex of SARS-CoV-2 spike RBD and cilgavimab (PDB: 7L7E) [9] was used. To predict the interaction between BA.2 spike RBD and cilgavimab, the spike RBD was replaced with the BA.2 spike mutants. The distance between residues was measured using PyMOL.

## 3. Results

**Figure 2:**
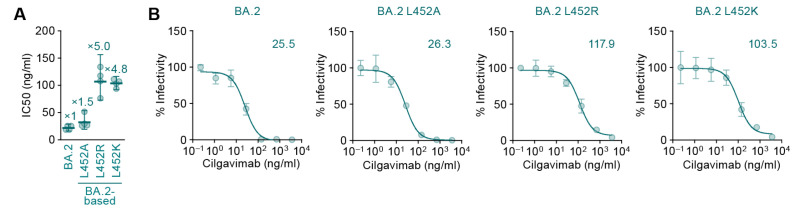
Neutralization sensitivity of the SARS-CoV-2 Omicron BA.2-based mutants to cilgavimab. (**A**) Pseudovirus neutralization assay. Assays were performed using pseudoviruses harboring the SARS-CoV-2 spike proteins of Omicron BA.2 and its derivatives (the BA.2 spike bearing L452A, L452R, or L452K, respectively). The assay was performed in triplicate at each concentration of cilgavimab to determine the IC50 value (ng/mL), and the assay was independently performed four times. Each dot represents one IC50 value, and the mean and 95% confidential interval is shown. The numbers indicate the fold changes of resistance versus BA.2. (**B**) Representative neutralization curves of the assay are shown. The numbers in the panels indicate the IC50 (ng/mL).

To address how the BA.2 spike L452R substitution renders resistance to cilgavimab, we performed a neutralization assay using cilgavimab and the BA.2 spike-based pseudoviruses. Consistent with previous reports [7,8], the L452R substitution significantly increased resistance to cilgavimab (*p* < 0.0005 by Student’s *t*-test) (Figure 2A). A previous report showed that the L452 residue of the BA.2 spike is located in the binding site of cilgavimab [9]. To reveal how the L452R substitution results in the resistance to cilgavimab, we prepared structural models based on a co-crystal structure of the SARS-CoV-2 spike RBD and cilgavimab (PDB: 7L7E) [9]. Our structural model showed that the L452 residue in the BA.2 spike was 4.4 Å apart from the S33 residue in the light chain of cilgavimab (Figure 1A). On the other hand, the estimated distance between the spike R452 residue and the S33 residue of cilgavimab was 1.7 Å, and the spike R452 residue exhibited steric hindrance with the S33 residue of cilgavimab (Figure 1C). These structural observations suggest that the higher resistance of BA.2 L452R to cilgavimab (Figure 2A) is attributed to the steric hindrance caused by the side chain of the amino acid position at 452. To address this possibility, we prepared two structural models based on the BA.2 spike: one bears the L452K substitution, which possesses a bulky side chain (K) similar to R, while the other bears the L452A substitution, which does not possess side chain. As expected, the estimated distance between the spike K452 and the S33 of cilgavimab was 2.0 Å (Figure 1D), suggesting that the L452K substitution also exhibits steric hindrance with the S33 residue of cilgavimab. On the other hand, the estimated distance between the spike A452 and the S33 of cilgavimab was 6.6 Å (Figure 1B), suggesting that the L452A substitution does not exhibit steric hindrance with the S33 residue of cilgavimab. In fact, a neutralization assay using the pseudovirus with BA.2 L452K or BA.2 L452A and cilgavimab showed that the L452K substitution affected the resistance to cilgavimab (*p* < 0.0001 by Student’s *t*-test), although the L452A substitution did not (Figure 2). Altogether, these results suggest the possibility that the BA.2 L452R substitution causes steric hindrance between spike and cilgavimab and results in the evasion from neutralization.

## 4. Discussion

Here, we demonstrated that the increased resistance of the BA.2 spike L452R to cilgavimab is potentially due to the steric hindrance between the spike binding interface with cilgavimab. The guanidine group of arginine is a hydrogen bond donor [15]. On the other hand, the hydroxyl group of serine can be both a hydrogen bond donor and a hydrogen bond acceptor [15]. Therefore, the side chain of the spike R452 and the S33 of cilgavimab have the possibility to form hydrogen bonds. However, our structural analysis showed that the distance between the two residues is too adjacent to form hydrogen bonds (Figure 1), implying that the spike L452R substitution of BA.4/5 contributes to resistance to cilgavimab by steric hindrance. Our data explain how the L452R substitution exhibits resistance to cilgavimab [8]. A previous study showed that the spike L452R substitution confers resistance not only to cilgavimab but also to other therapeutic mAbs, such as etesevimab and bamlanivimab [9]. Moreover, the spike L452R substitution was observed in other major SARS-CoV-2 variants, such as the Delta and Epsilon [16]. Therefore, our results suggest the importance of developing therapeutic mAbs capable of inhibiting the infection of SARS-CoV-2 variants bearing spike L452R substitution. Since the L452R substitution attributes to evasion from various therapeutic mAbs, continuous monitoring of the substitution of this residue should be important.

## 5. Conclusions

Although the SARS-CoV-2 Omicron BA.4 and BA.5 variants are resistant to cilgavimab, the mechanism of this resistance remains unclear. Here we show the possibility that the L452R-mediated resistance to cilgavimab is attributed to the steric hindrance between cilgavimab and its binding interface on the spike.

## Data Availability

Not applicable.
